# (Neuro) Peptides, Physical Activity, and Cognition

**DOI:** 10.3390/jcm9082592

**Published:** 2020-08-10

**Authors:** Juho Autio, Ville Stenbäck, Dominique D. Gagnon, Juhani Leppäluoto, Karl-Heinz Herzig

**Affiliations:** 1 Institute of Biomedicine, Medical Research Center, Faculty of Medicine, University of Oulu, Oulu University Hospital, 90220 Oulu, Finland; Juho.Autio@mail.com (J.A.); Ville.Stenback@oulu.fi (V.S.); ddgagnon@laurentian.ca (D.D.G.); juhani.leppaluoto@oulu.fi (J.L.); 2 Biocenter Oulu, 90220 Oulu, Finland; 3 Laboratory of Environmental Exercise Physiology, School of Human Kinetics, Laurentian University, Sudbury, ON P3E 2C6, Canada; 4 Center of Research in Occupational Safety and Health, Laurentian University, Sudbury, ON P3E 2C6, Canada; 5 Department of Gastroenterology and Metabolism, Poznan University of Medical Sciences, 60-572 Poznan, Poland

**Keywords:** (neuro) peptides, physical activity, cognition

## Abstract

Regular physical activity (PA) improves cognitive functions, prevents brain atrophy, and delays the onset of cognitive decline, dementia, and Alzheimer’s disease. Presently, there are no specific recommendations for PA producing positive effects on brain health and little is known on its mediators. PA affects production and release of several peptides secreted from peripheral and central tissues, targeting receptors located in the central nervous system (CNS). This review will provide a summary of the current knowledge on the association between PA and cognition with a focus on the role of (neuro)peptides. For the review we define peptides as molecules with less than 100 amino acids and exclude myokines. Tachykinins, somatostatin, and opioid peptides were excluded from this review since they were not affected by PA. There is evidence suggesting that PA increases peripheral insulin growth factor 1 (IGF-1) levels and elevated serum IGF-1 levels are associated with improved cognitive performance. It is therefore likely that IGF-1 plays a role in PA induced improvement of cognition. Other neuropeptides such as neuropeptide Y (NPY), ghrelin, galanin, and vasoactive intestinal peptide (VIP) could mediate the beneficial effects of PA on cognition, but the current literature regarding these (neuro)peptides is limited.

## 1. Introduction

Cognition is a complex system encompassing processes such as episodic memory, working memory, executive function/inhibition, spatial learning, language/vocabulary comprehension, processing speed, and language/reading decoding [1]. This is thought to be generated by association connections between different cerebral cortex regions [2,3]. Inhibitory and stimulatory inputs strengthen (long term potentiation) or weaken (long term depression) the synaptic activities, rendering the connectivity a very dynamic process. This synaptic plasticity is influenced by electrical synapses which have greater speed in transmission and synchronization of groups of neurons, and chemical synapses with amino acids (gamma-aminobutyric acid, glutamate, glycine), amines (acetylcholine, dopamine, histamine, norepinephrine, serotonin), or peptides as neurotransmitters (neuropeptides YY, vasoactive intestinal polypeptide, enkephalins, substance P, cholecystokinin). Changes in synaptic plasticity can be short lived from milliseconds to years. Short lived forms include facilitation, augmentation, and potentiation which enhances neurotransmitter release. These dynamic changes represent the molecular basis for learning and memory [4]. This synaptic plasticity can be influenced by several factors e.g., aging, diseases (obesity, diabetes, hypertension, dyslipidemia), toxins (smoking and alcohol), and exercise. Aging has been estimated to trigger performance decline with an incidence of mild cognitive impairment of 21.5–71.3 per 1000 person-years [5]). Cortical thickness and subcortical volume are shrinking 0.5–1% annually as a morphological sign of the cognitive decline [6] with plaques and axonal degeneration [7]. Dementia is diagnosed, when the acquired cognitive impairment has become severe enough to compromise social and/or occupational functioning with increasing prevalence.

Worldwide, around 50 million people have dementia and, with one new case every three seconds, the number of people with dementia is set to triple by 2050 [8].

Physical activity (PA) has been used and prescribed as medicine in the treatment of various diseases including psychiatric, neurological, metabolic, cardiovascular, and pulmonary diseases [9]. An alarming sign is that more than 1.4 billion adults do not get the recommended levels of physical activity [10]. PA has been clearly shown to improve cognitive function across the lifespan in animal studies [11,12] and in humans over 50 years regardless of their cognitive status [13]. The effects of PA include enhancement of cognitive abilities and decelerating the age-induced decline of cognitive function [14,15]. In adults, there is a positive association between PA, hippocampal [16,17,18,19,20], and pre-frontal cortex gray matter volume [16]. The positive effects of physical activity include increases angiogenesis, formation of new dendritic spines [21], neurogenesis in the dentate gyrus of hippocampus [14,22,23], synaptic plasticity via long term potentiation, hippocampal volume, learning, and memory [20,24].

**Figure 1:**
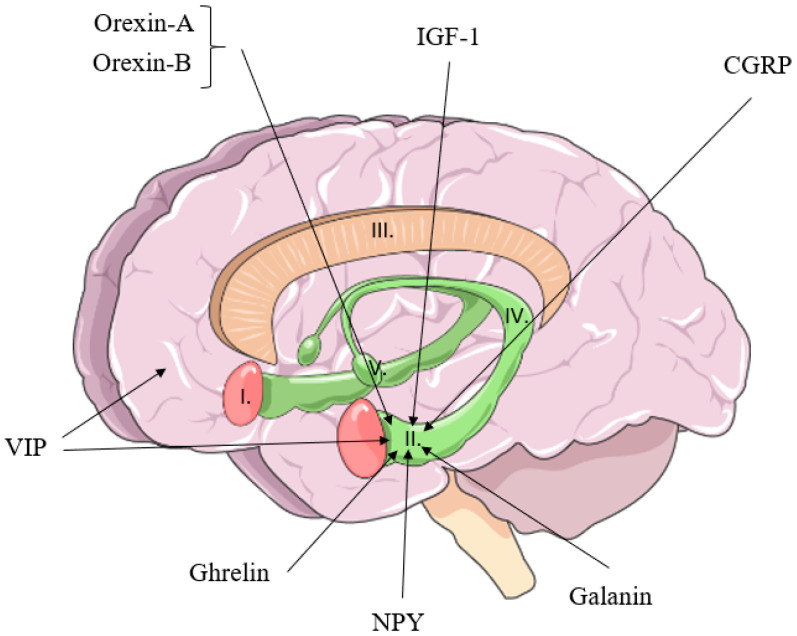
Summary of (neuro)peptides released by physical activity on cognition. I: Amygdala. II: Hippocampus. III: Corpus callosum. IV: Fornix. V: Mammillary body. NPY: Neuropeptide Y IGF-1: Insulin-like growth factor 1 CGRP: Calcitonin Gene-Related Peptide VIP: Vasoactive intestinal peptide.

PA affects production and release of several (neuro)peptides secreted from peripheral tissues like muscle, adipose tissue, and liver or by the brain itself, targeting receptors inside the central nervous system (CNS). Physical activity can be aerobic, anaerobic, or resistance activity with acute or chronic duration. This review will provide a summary the association between PA and cognition with a focus mostly on humans and the role of (neuro)peptides (Table 1, Table 2 and Table 3 and Figure 1). Duration and type of exercise are mentioned in the Tables. We define peptides as molecules with less than 100 amino acids (aa) [25]. Thus, we excluded growth factors such as brain-derived neurotrophic factor (BDNF; 119 amino acids) and vascular endothelial growth factor (VEGF; 121, 145, 165, 183, 189 and 206 amino acids), which have previously been thoroughly reviewed [26,27]. The association between PA and myokines have been recently discussed [28].

## 2. Insulin Growth Factor 1 (IGF-1)

The physiological role of insulin growth factor 1 (IGF-1) is complex, and several different variables such as binding, isoforms, compartmentalization, and cellular signaling pathways contribute to the effect of IGF-1 in the body [56,57]. IGF-1 receptors have the highest density in the hippocampus [58,59,60,61], parahippocampal gyrus, and amygdala [58]. IGF-1 plays an important role in growth and differentiation of neurons, neurotransmitter synthesis [62], stimulating neurogenesis in both the hippocampus [63] and hypothalamus [64]. Thus, cognition, learning and memory are likely influenced by serum IGF-1 [65,66]. Additionally, IGF-1 has been suggested to be a mediator of beneficial effects of exercise to cognition in several human studies (Table 1) [67].

Although the exact mechanisms remain unknown, the evidence obtained from rodent studies using radiolabeled IGF-1 suggests that IGF-1 crosses the blood-brain barrier via specific transcytosis [68]. Animal studies suggest that PA increases the uptake of serum IGF-1 to brain via the blood-CSF pathway utilizing specific groups of neurons located in different brain regions like the thalamus, pyramidal cell layer of hippocampus, and striatum [66].

### 2.1. Centrally and Peripherally Released IGF-1

Animal studies have shown that IGF-1 is produced centrally in regions of the brain where post-natal neurogenesis occurs, specifically in cerebellum, olfactory bulb, and hippocampus [69]. IGF-1 is also produced in liver tissue [70,71]. The plasma levels peak at adolescence and decline thereafter [72]. Females had higher levels at the age of 21–30 compared to males but declined afterwards faster with no gender difference in the age group of 31–40 years. Liver IGF-1 gene disrupted mice [71] had reduced cognitive abilities, specifically spatial-recognition, compared to control group [73]. The gene impairment resulted in ~60% lower serum IGF-1 levels, but their IGF-1 mRNA levels in the brain tissue remained normal. Moreover, chronic infusion of hIGF-1 into gene disrupted mice increased the cognitive abilities of mice but not in controls. The authors concluded that liver-derived circulating IGF-1 could play an important role in cognition of mice [73], however, they could not exclude the possibility of other endocrine/metabolic abnormalities in these gene-disrupted mice causing the cognitive deficits. Similar results were obtained by Svensson and colleagues [74] who used a liver-specific, inducible inactivation of the IGF-I gene via the Cre-LoxP conditional knockout system (LI- LI-IGF-I−/− mice) in adult mice and observed memory and cognition deficits as the mice grew older (12–15 months). In mice and rats, blocking the IGF-1 uptake to brain with anti-IGF-1 antibody prevented the neuroprotective effects of exercise [63,75]. IGF-1 receptor expression increased with age in the hippocampus [76]. Intraperitoneously injected human IGF-1 into young (4 months old) and old (24 months old) mice caused a higher increase in the CSF in the old mice [77]. Furthermore, IGF-I-induced signaling via the brain IGF-I receptor was significantly diminished in the aged animals, suggesting receptor resistance in the aging process.

### 2.2. IGF-1 and Cognition

Elevated serum IGF-1 levels were associated with improved cognitive performance [31,35,78,79,80,81,82]. Higher free plasma IGF-1 levels were correlated with better cognitive performance in middle-aged human males [83] and elevated (plasma/serum) IGF-1 levels have been associated with improved cognitive performance in older men [84,85]. Decreased serum levels of IGF-1 below 9.4 nmol/l were negatively associated with cognitive performance [86]. In elderly, raised IGF-1 levels resulted in shorter reaction times in tasks evaluating working memory and better ability to recruit prefrontal areas compared to subjects with lower peripheral IGF-1 levels [79].

Ferro and colleagues investigated the effect of PA on executive function in a longitudinal study, following 303 subjects from 13 to 42 years of age [32] (Table 1). PA levels during adolescence were associated with executive function during adulthood in males but not in females. Males who were more active during adolescence performed better in cognition tests, yet there was no association with IGF-1 levels in either sex. The authors suggested that only males, but not females, reached a threshold of activity that promoted improved cognition. Moreover, males and females may differ in the physiological responses to intensive endurance training as adult females had blunted maximal oxygen consumption (VO_2max_) increases after one-year endurance training compared to adult males [87]. Similarly, Lee and colleagues reported that aerobic exercise significantly improved adolescents’ performance on tests measuring frontal and temporal lobe function [34]. Interestingly, they found no significant association between exercise dose and plasma IGF-1 levels.

### 2.3. IGF-1 and Age

The IGF-1 levels have been found to decrease with age [69,88,89]. Younger men had higher resting IGF-1 levels and increased IGFBP-3 levels compared to older men [90]. In elderly males, resistance exercise group improved reaction times only, along with elevated levels of serum IGF-1 compared to the control group [91]. In elderly females, martial arts practice (taekwondo) resulted in increased serum resting levels of neurotrophic growth factors (IGF-1, BDNF, and VEGF) [31]. In addition, the cognitive abilities were improved, possibly due to increased serum levels of neurotrophic growth factors [31]. However, there was no information provided on estrogen supplementation as a possible confounding factor. These results indicate that PA could attenuate age-related decline of neurotrophic growth factors, including IGF-1, and therefore could ameliorate the age-related decrease in cognitive abilities.

### 2.4. IGF-1 and Gender

In men with mild cognitive impairment Baker and colleagues reported an increase in IGF-1 plasma levels and improved performance in the Trails B test [92] in response to aerobic exercise, but not in other cognitive tests [29]. Women with mild cognitive impairment improved on multiple cognitive tests in response to exercise but without changes in IGF-1 plasma levels [29]. The authors suggested that the observed gender differences might be related to gender-based differences in gluco-metabolic and hypothalamic-pituitary-adrenal axis responses to aerobic exercise. Unfortunately, the status of estrogen supplementation of women was not mentioned in the study. In elderly men (65–75 years; no information was given on their training status before entering the study), resistance training (high and moderate intensity) improved cognition and raised serum IGF-1 levels compared to control subjects [30] (Table 1). Strength training did not change peripheral IGF-1 levels in older post-menopausal women (64 ± 3 years) [93]. The subjects in the later study kept themselves physically fit via various recreational low-intensity physical activities such as walking, jogging, cross-country skiing, aerobics, or biking, but none of the subjects had any background in regular strength training.

### 2.5. IGF-1 and Fitness

Significantly elevated resting levels of IGF-1 were found in trained adolescents compared to sedentary controls [94]. The blood samples were taken in the morning after an overnight fast. Similarly, Nindl and colleagues found [95] that IGF-1 levels were positively associated with aerobic fitness in men, confirming results published by Pareja-Galeano and colleagues [94]. However, other studies reported no association between aerobic physical capacity and IGF-1 levels in adults [89] and in adolescents [34]. This suggests that previous physical fitness might be a significant confounding factor in the body’s IGF-1 response to further physical activity.

### 2.6. IGF-1 and Physical Activity

The type of PA (resistance or aerobic exercise) and its frequency (acute or chronic) influence IGF-1 levels. Even short 10 min high- or low-intensity exercise bouts elicit increases in serum IGF-1 concentrations [96]. Strength training induced acute increases [30,97,98,99,100] and raised resting serum IGF-1 levels [101]. However, other groups studying strength training reported no change in peripheral IGF-1 levels in 18–30 years old [33], in 18–25 years old [102], and in 30 and 62 years old [90] healthy humans. In athletes, short- or long-time exercises had no significant effect on circulating IFG-1 concentrations [103,104]. Even decreased basal plasma IGF-1 concentration after moderate endurance and strength training in healthy sports students (age 22–23 years) [105] or after strength training to repetition failure in 24 years old Basque ball players with 12.5 ± 5 years of regular training and competition experience have been reported [106]. Aerobic (endurance-type) training lowered serum IGF-1 and IGFBP-3 levels [107].

Winker and colleagues suggested that endurance training might help to maintain cognitive abilities in elderly persons [37]. However, IGF-1 levels in their cross-sectional cohort study were not associated with the amount of exercise. In healthy middle-aged women, neither resistance training nor aerobic exercise significantly increased IGF-1 levels compared to the control group [108].

Energy balance might play a role in the effects of physical activity to IGF-1 levels [102]. In young healthy men, age 18–25 years., peak O_2_ uptake (VO_2_) of 35–45 mL O_2_/kg/min, a negative energy balance combined with exercise training resulted in substantial decrease of free and total IGF-1. IGF-1 levels remained unchanged in the group with positive energy balance and exercise training. In the negative energy balance group IGF-1 and IGFBP-1 levels returned to normal within one week after the exercise program and diet. This suggests that the energy balance might be a key modulator of the effects of exercise on IGF-1 levels [35,102].

### 2.7. Associations between Physical Activity, IGF-1, and Cognition

IGF-1 mediates several beneficial effects of exercise on learning, depression, stimulation of angiogenesis, and hippocampal neurogenesis [67,109,110,111].

Cassilhas and colleagues [30] reported that both aerobic and strength training increased cognitive abilities. There is evidence indicating that acute and chronic aerobic exercise increases peripheral BDNF levels [26]. As BDNF is a crucial mediator in brain health [26], this could explain the increase in cognitive abilities caused by both strength and aerobic exercise. IGF-1 and BDNF signaling share downstream pathways [112]. They both led to improvements in brain health and cognition [67,109] and thus differentiating the effects of IGF-1 and BDNF might not be straightforward. For example, blocking the IGF-1 receptor resulted in decrease of exercise induced elevation of serum BDNF, BDNF mRNA and pro-brain-derived neurotrophic factor protein levels in mice [113]. Conversely, BDNF blocking inhibited the effect of physical activity to IGF-1 mRNA [114]. The circulating IGF-1 system seems to adapt to physical activity and this could, at least partly, mediate the beneficial effects of exercise to cognition.

### 2.8. IGF-1 and Intervention Studies

Intervention studies provide strong evidence for the effects of physical activity on memory and cognition. Activity interventions have shown that physical activity leads to increase of new neurons in the hippocampus of adult rats and the effects were mediated by IGF-1 [63,66]. In addition, intervention studies on sedentary elderly humans found improved cognitive abilities and increased IGF-1 levels in response to aerobic [29,31], moderate, and high intensity resistance exercise [30,36]. However, Goekint and colleagues could not show these associations in 18–30 years old untrained subjects [33]. An intervention study in teenagers found an association between regular aerobic exercise and cognitive abilities without increased IGF-1 serum levels [34].

### 2.9. IGF-1 and Prospective Trials

There is an ongoing longitudinal follow-up study to elucidate the roles of IGF-1, PA and cognition [115]. The study follows 500 preschool children (3.5–5.5 years old) over the span of 15 years with six triennial assessment time periods which will include 7-day physical activity measurement with accelerometers, cognitive development evaluation, anthropometric and physical fitness assessments, plasma IGF-1 and BDNF level measurements, and retrospective questionnaires.

Clearly, additional studies are needed to elucidate which is the best PA regime (aerobic, resistance, acute, chronic) to induce/maintain IGF-1 levels and to further decipher the molecular pathways that mediate its potential beneficial effects PA on neurogenesis, synaptic plasticity, and cognition.

## 3. Orexins

Orexins (orexin-A/hypocretin-1 and orexin-B/hypocretin-2) are peptides produced by neurons in the perifornical area and lateral hypothalamus [116,117]. These neurons project to multiple locations in the brain such as the thalamus and the brainstem. Orexin neurons have several physiological roles in feeding, energy homeostasis, sleep/wake state, rewards systems, mood, and cognition [118,119,120]. Orexins are suggested to have beneficial effects to hippocampal neurogenesis improving memory abilities, spatial learning and mood [119]. Conversely, orexin deficiency can lead to impaired cognitive, learning, and memory abilities [119].

There are only few investigations on the orexin system, PA, and cognition in humans, but animal studies have demonstrated beneficial effects of PA on plasma orexin-A concentrations and cognition. Messina and colleagues [121] reported that PA using a calibrated mechanically braked cycle ergometer (at 75 W for 15 min) increased orexin-A levels in plasma of humans from 2610 ± 187 pg/mL to a maximum level (3589 ± 197 pg/mL) within 30 min after exercise. While relative changes in orexin blood levels may be due to experimental differences, absolute levels are still unclear as commercial kits and extraction protocols differ significantly compared to an in-house generated antibody with plasma levels in the range of 1–16 pg/mL [122].

Orexin-A, but not Orexin-B, readily crosses the blood–brain barrier (BBB) [123]. Zhao and colleagues reported that after intracerebroventricular (ICV) injection of orexin-A in rats with learning and memory deficiencies improved spatial learning and memory [124]. The ICV orexin-A administration increased cell proliferation and neurogenesis in dentate gyrus compared to control rats. During aging orexin neurons significantly decreased in rodents [125,126,127].

These findings suggest that orexin-A might mediate beneficial effects of PA on cognition. However, it is currently unknown to which extent these results can be extrapolated to humans.

## 4. Ghrelin

Ghrelin is a growth-hormone releasing peptide, mostly produced in the stomach [128]. Ghrelin is high in the inter-digestive phase, initiating food intake [129] and may be altered in obesity [130]. Interestingly, caloric restriction has been shown to improve memory in elderly subjects [131]. Animal studies have shown that ghrelin, which crosses the BBB [132,133], has neurogenic effects on the hippocampus in adult brains [134] and ghrelin levels were associated with cognitive function [135].

ICV injections of ghrelin resulted in increased memory retention in rats [136]. In addition, administration of ghrelin into hippocampus [137] and basolateral amygdala [138] of rats resulted in enhanced learning and memory. Ghrelin receptor knockout mice had decreased spatial learning abilities [139]. Rats treated with intravenous (iv) acyl-ghrelin injections had an increased neurogenesis and better spatial memory [140]. Intravenous administration of ghrelin into adult mice stimulated hippocampal neurogenesis without an effect on spatial memory [141]. Interestingly, local administration of ghrelin into the CA1 region of the hippocampus had no effect on neurogenesis but impaired spatial memory formation [141].

Exercise types affect peripheral ghrelin levels [142]. The energy status is especially important when determining ghrelin levels. All cited studies investigated the subjects in their fasted state with blood sampling before and immediately after the exercise. The exception were subjects studied by Deighton et al., in which the subjects consumed a breakfast before exercise and blood sampling [38] (Table 2).

In humans, single sessions of aerobic exercises resulted in lower plasma concentrations of acylated ghrelin [38,39,40,41,42] while other authors did not find any change in plasma acylated ghrelin levels [43,44]. Single resistance exercise sessions suppressed [40,44,45] or had no effect on the plasma acylated ghrelin levels [42,44] (Table 2). Long-term aerobic training programs slightly increased acylated ghrelin levels at rest [46]. Other groups did not find association between aerobic or resistance training and plasma active ghrelin levels [47,48].

In conclusion, animal studies demonstrate a clear effect on cognitive functions, yet studies in humans are inconclusive due to the strong regulation by the energy status which changes by PA.

## 5. Neuropeptide Y (NPY)

Neuropeptide Y (NPY) is a 36 amino acid peptide exclusively produced in the neural tissues and widely expressed in the CNS [143,144]. NPY has five G-(guanine nucleotide-binding) coupled receptors (Y1, Y2, Y4, Y5, Y6; the Y6 receptor seems not be expressed in primates) which can be found throughout the central nervous system [145]. The effects of NPY on cognition are highly region specific [145,146]. NPY crosses the BBB in a non-saturable manner by diffusion [147].

In rats, wheel running resulted in an increased hippocampal NPY mRNA expression [148] and NPY content in paraventricular, dorsomedial, and ventromedial hypothalamic nuclei [149]. In addition, hypothalamic NPY concentrations increased by 30–70% with intense physical exercise (wheel running, 40% of daily energy intake) in the arcuate and dorsomedial nuclei and medial preoptic and lateral hypothalamic areas of rats [150]. In humans, plasma levels of NPY increased parallel with blood pressure, heart rate, and catecholamines during exercise [151] and from sympathetic nerve endings around blood vessels and within the myocardium with an increased heart rate [51] (Table 3). A significant rise in plasma NPY was reported after 10 min of ergometer training (until exhaustion, 50W increase in load with every 3 min) and stayed elevated for 25 min. after exercise [152]. A single prolonged vigorous exercise (marathon swimming) or a 2-week of intense training significantly elevated plasma NPY levels [52,53].

NPY appears to be a mediator of mnemonic processes [146,153] with very region specific actions in CNS ranging from enhanced memory [154] to no effect [154,155] and to amnesic effect [154]. NPY, PYY, and the Y_1_ agonists ameliorated learning impairments caused by dizocilpine in mice, suggesting NPY and calcitonin gene-related peptide (CGRP)-related peptides (see below) may be involved in modulating the cognitive processes [156]. In contrast, Karl and colleagues reported no difference in learning abilities between NPY gene-deficient and wild type mice, using passive avoidance testing to investigate learning and memory abilities [157]. The passive avoidance test is dependent on the animal’s ability to experience pain, while NPY KO mice might have impaired nociception [158]. Furthermore, NPY has been shown to be associated more with memory consolidation and recall than acquisition in mice [159]. Overexpression of NPY in the hippocampus of rats led to partial inhibition of cognitive function by delaying the learning process but did not prevent memory acquisition [160]. In addition, age might be a confounding factor for NPY on learning since hippocampal overexpression of NPY led to impaired spatial learning in young rats [161], but not in old rats [162].

In summary, there is strong evidence that NPY modulates neuroplasticity and learning [163]. How physical activity via the release of NPY from peripheral nerve endings will modify neuroplasticity and learning is currently unknown.

## 6. Galanin

Galanin is a 29 amino acid peptide isolated from porcine intestine [164]. The human form consists of 30 amino acids. It does not cross the BBB [165]. Therefore, positive effects on cognitive function by galanin must come from endogenous production of the peptide within the CNS. Animal studies have shown that rats with a higher running capacity have elevated expression of galanin mRNA in the locus coerulus as a response to the exercise [166].

The physiological functions of galanin are mediated by three receptors (GALR1-3) [167] and have been nicely summarized recently [168,169]. GALR can form dimer and heterodimers (e.g., %-HT1A or NPY Y2), changing the postreceptor signaling. Animal studies suggested that galanin may act as both excitatory and inhibitory neuropeptide on cognition and its effects may be mediated by other mechanisms, e.g., release of endogenous excitatory amino acids or acetylcholine [170,171,172,173,174].

In the hippocampus galanin receptors GALR1 and GALR2 are differently and species dependently expressed. Thus, the effects of the administrated galanin on cognition are both site and dose dependent [153,174,175,176]. In rats, galanin is modulator of cholinergic transmission [177] while in monkeys and humans galanin is not co-localized in acetylcholine neurons of the nucleus basalis of Meynert [178].

In rodents, ICV administration of galanin led to an impairment of memory related tasks [174,176], and acquisition, but not retrieval of partial memory in rats [179]. Memory consolidation was also negatively affected by ICV administration of galanin [180]. Infusion of high dose of galanin into the ventral and dorsal hippocampus of rat resulted in acquisition deficit in spatial task performance [172,173,181,182], while infusion of galanin to the ventral CA3 region, which expresses GALR1, affected performance in Morris water maze task dose dependently: 3 nmol infusion caused a slight impairment, a 1 nmol infusion improvement, and a 6 nmol dose failed to cause any change [172]. Infusion of galanin to medial septal area of rats improved spatial acquisition, while infusion of galanin into the dorsal CA1 region, devoid of galanin receptors, did not influence spatial learning and memory performance [173]. In summary, these results suggest that galanin plays a significant role in cognition and its effects are dose and region specific.

Transgenic mice overexpressing galanin in hippocampus showed selective memory deficits [183]. However, galanin overexpression in other strains of transgenic mice did not cause any memory deficits [184]. Wrenn and colleagues [185] did not find consistent correlations between the performance in multiple cognitive tasks in mice with galanin overexpression in hippocampus [185]. The authors suggested the possibility of a threshold that would need to be crossed to initiate the performance deficits on the memory tasks [185]. In addition, the effect of galanin might be age dependent since 10-month galanin knockout mice showed impairment in memory tasks, whereas no difference between 4-month old knockout and wildtype mice was observed [186]. Similar results have been obtained in 19-month-old galanin-overexpressing mice with memory deficits in water maze test compared to the 9-month-old galanin-overexpressing mice [187]. Interestingly, GALR1-knockout mice showed normal performance in cognitive tasks (social transmission of food preference and Morris water maze test) compared to the wild type mice, with the exception of cued fear conditioning, in which homozygous GALR1 KO mice showed impaired performance [188].

In summary, most of our current knowledge comes from rodent work. Human studies are sparse and more focused on diseases like major depressive [189] disorder or Alzheimer’s Disease [190]. The translation of animal studies to the human physiology is not straightforward because of species differences.

## 7. Vasoactive Intestinal Peptide (VIP)

Vasoactive intestinal peptide (VIP) is expressed widely in the periphery (gastrointestinal tract, heart, lungs, thyroid, urinary bladder, kidney, and genital organs) and CNS [191,192,193]. In the CNS, VIP and its receptors are present in several brain regions associated with memory and learning such as hippocampus, amygdala, and cortex [175,194]. VIP crosses the BBB unidirectionally from blood to the brain tissue by transmembrane diffusion [195]. In mice, hippocampal neurogenesis declines with age [196], as well as the number of neurons expressing VIP, NPY, and somatostatin [197].

Chaudhury and colleagues found a normal behavior in VIP deficient mutant mice compared to control mice, except in tests 48 h after the contextual fear conditioning with a significant reduction in memory recall [198]. In addition, amnesic effects of VIP have been reported after injections of non-physiological doses of VIP to lateral cerebral ventricles [199] and hippocampi [200,201]. In contrast, intraperitoneal, subcutaneous, or ICV injection of VIP improved the scopoloamine-induced amnesia of rats [202]. Similar anti-amnesic effects of VIP were reported by Glowa and colleagues, demonstrating that ICV infusion of VIP ameliorated the amnesia induced by gp120 (external envelope glycoprotein of the human immunodeficiency virus) in Morris water maze test [203].

Injections of VIP antagonists to pregnant mice resulted in severe microcephaly fetuses’ brain [204], in reduced sociability and impairment of cognitive function [205,206]. Animal studies have shown that pharmaceutical blockade of VIP during early post implantation resulted in impaired cognition in the offspring [207]. Transgenic mice carrying a chimeric VIP gene driven by the polyoma promoter had a 20% reduction in VIP content in the brain, leading to cognitive deficiencies [208].

In humans, plasma VIP levels increased in response to physical activity. MacLaren et al. observed significantly elevated plasma VIP levels during 90 min of running in endurance athletes and hockey players with a greater change in the latter to baseline levels [209]. Rolandi et al. reported significantly higher plasma VIP levels after both exhaustive and submaximal exercise in marathon runners [210]. Both exercise until exhaustion over 16 min and mild PA lasting over 3 h elevated plasma VIP levels significantly [50,211] (Table 3), indicating that plasma VIP increases are time and intensity dependent. In Norwegian military academy cadets VIP levels significantly increased in a 5-day training program with prolonged physical exercise with approximately 35% of their maximal oxygen uptake, caloric deficiency, and sleep deprivation [49,212]. Calorie-compensation during the prolonged exercise or glucose infusion ergometer exercise testing lowered the increase of VIP plasma levels [49].

In summary, VIP has important effects on synaptic plasticity, behavior, and cognition and increases with PA. To which extent PA induced VIP release changes cognition is still unclear. Consequently, further research on the effect of VIP on cognition is warranted.

## 8. Calcitonin Gene-Related Peptide (CGRP)

Calcitonin gene-related peptide (CGRP) is a 37 amino acid neuropeptide produced in the peripheral and central nervous system [213]. Plasma levels are very low and thought to be the result of spillover from the perivascular nerve endings by sympathetic agonists with a rapid degradation [214] or via the activation of sensory fibers [215]. Subcutaneous capsaicin, a transient receptor potential cation channel subfamily V member 1 (TrpV1) activator in sensory nerve terminals, significantly increased the hippocampal tissue levels of CGRP, IGF-I, and IGF-I messenger RNA (mRNA) in wild-type (WT) but not in CGRP-/-mice [216]. Furthermore, significant stimulation of neurogenesis was detected in the dentate gyrus after capsaicin administration in WT but not in CGRP-/-mice. The capsaicin-induced improvement of the spatial learning was reversed by administration of an anti–IGF-I antibody and the CGRP receptor antagonist CGRP (8–37). CGRP is not likely to cross BBB [217], but treatment with capsaicin resulted in increased permeability [218]. Intraperitoneal injections of ghrelin increased hippocampal CGRP, IGF-1, and IGF-1 mRNA expression levels in wild-type (WT) with but not in CGRP-/-knockout mice [219]. The author suggested that ghrelin stimulates neurons in circumventricular organs, increasing the release of CGRP in the hippocampus, which in turn stimulates the production of IGF-1 with the subsequent improvements in cognition.

In humans, aerobic exercise raised CGRP plasma levels in a dose dependent manner but no significant elevations of CGRP resting levels were observed [54,55] (Table 3). Fitness level did not affect resting CGRP plasma levels [55].

The role of CGRP in cognition has recently been summarized in detail [175]. Currently, little is known about the effect of exercise on CGRP levels in the CNS and its positive effects on cognition.

## 9. Other (Neuro-)Peptides

Several other peptides influence the cognitive function and their effects have been comprehensively reviewed by Borbély and colleagues [175]. Tachykinins (neurokinins A and B, substance P), somatostatin and opioid peptides were excluded from this review since they were not affected by PA.

## 10. Conclusions and Future Directions

This review attempted to summarize the current knowledge of the effects of PA induced peptide release on cognition. Cognition is a complex system with changes in synaptic plasticity influenced by a magnitude of transmitters (amino acids, amines peptides) and time dependent (milliseconds to years). Furthermore, physical activity as “bodily movements produced by the skeletal muscles” has a wide range with planned and repeated physical exercise to improve or maintain bodily functions. In addition, the physical exercise can be structured into aerobic/endurance or anaerobic/resistance exercise in an acute or chronic setting. With these differences in mind it has been shown that PA has a clear beneficial effects on brain health and cognition, yet the current data base is limited in terms of peptide mediators (Table 1, Table 2 and Table 3 and Figure 1).

There is considerable scientific evidence that elevated serum IGF-1 levels are associated with improved cognitive performance. However, some studies found no association between peripheral IGF-1 levels and cognition. These discrepancies might be due to confounding factors in the different trials such as age and fitness, or the maintenance of energy balance during treatment or intervention. Moreover, PA increased peripheral IGF-1 in untrained, older subjects, after the decline of the endogenous levels. It is therefore likely that IGF-1 plays a role in PA induced improvement of cognition. Other neuropeptides, such as NPY, ghrelin, galanin, and VIP, could mediate beneficial effects of PA on cognition. However, most studies were performed in animals (rodents) and it is very difficult to estimate to which extent these results could be translated towards humans. Additional modulating factors are aging, gender, and diseases. The urgency for remedies to alleviate our cognitive decline is enormous in the light of, e.g., the huge economical costs cause by dementia for our aging societies, yet the scale and profile of the global dementia research funding has been sparse [220]. This has particularly been the case for physical activity interventions.

The current scientific data suggest that most likely we cannot expect to find the answer in one peptide or transmitter or in one PA setup but would need to carry out more carefully defined intervention, control confounding factors and individualize the intervention protocols (e.g., age, gender). This will be tedious, costly, and time consuming, but certainly rewarding in the long term.

## Figures and Tables

**Table 1:** Human studies on effects of physical activity on peptides and cognition: insulin growth factor 1 (IGF-1).

Reference and Study Design	Subjects	Target Area	Type of PA	(Neuro) Peptide	Main Results
RCT [29]	33 M and F Sedentary with mild cognitive impairment. 55–85 years old (mean age: 70 years)	Executive function and short-term memory	Acute aerobic exercise	IGF-1	For women, aerobic exercise improved cognitive performance. For men, aerobic exercise increased plasma IGF-1 levels and improved performance on trails B test.
RCT [30]	62 M Sedentary 65–75 years old	Executive function, short-term memory, attention and long-term episodic memory	Acute, moderate- and high-intensity resistance exercise	IGF-1	Moderate and high-intensity resistance training have equally beneficial effects on cognition. IGF-1 levels were higher in subjects compared to controls.
RCT [31]	37 F Sedentary 65+ years old	Selective attention, cognitive flexibility, and processing speed	Acute aerobic exercise	IGF-1	Taekwondo training may improve physical fitness and cognitive functioning in elderly females. IGF-1, VEGF and BDNF may mediate the latter effect.
Longitudinal study [32]	303 M and F Followed from age 13 to 42 years old	Executive functioning and visual-spatial memory	Physical activity and fitness were assessed annually between ages 13 to 16. At mean age 36, physical activity and fitness were assessed.	IGF-1	For males, there is a significant association between physical activity in adolescence and cognitive capabilities in adulthood. Such association was not found for females. IGF-1 was not found to have an intermediate role for either sex.
Controlled trial [33]	23 M and F Untrained 18–30 years old	Short- and mid-term memory	Regular resistance exercise	IGF-1	10 Week strength training period did not influence serum BDNF, IGF-1 or IGFBP-3 levels. There was no difference in short-term memory between experimental and control group. Mid-term memory was not improved after 10-week training program.
Between-group design [34]	91 M and F Healthy, both sedentary and physically active 14–18 years old	Selective attention, cognitive flexibility, processing speed, spatial memory, spatial memory and primary visual cortex function	Regular aerobic exercise	IGF-1	Regular aerobic exercise improves cognitive function in teens and these beneficial effects are associated with serum levels of BDNF and VEGF. No such association was found for IGF-1.
Cross-sectional study [35]	22 M and F 65–85 years old (median age: 77 years)	Temporo-spatial orientation, memory, attention, calculation capacity, language, and pragmatic praxia	Questionnaire about physical activity and performance	IGF-1	No association between physical activity and GH or IGF-1 levels were demonstrated. There is a direct relationship between cognitive function and IGF-1 plasma levels in aged subjects with cognitive impairment.
RCT [36]	60 M Exercise status was not mentioned 20–29 years old	Executive function, inhibitory control and attention	Acute resistance exercise	IGF-1	The beneficial effects of resistance exercise on cognitive function might be explained by changes in arousal state. The change could possibly be modulated by serum cortisol levels.
Cross-sectional study [37]	114 M and F Physically active 60+ years Old (mean age: 66 years)	Executive function, visuo- construction, concentration/attention, language, and memory	Regular aerobic exercise	IGF-1	Extensive aerobic training might be beneficial in maintaining cognitive function in older age. BDNF and IGF-1 was not found to be associated with duration of daily exercise and no differences in the basal levels of BDNF and IGF-1 between exercise and control group was found.

M, males; F, females; RCT, randomized controlled trial; PA, physical activity.

**Table 2:** Human studies on effects of physical activity on peptides and cognition: Ghrelin.

Reference	Subjects	Target Area	Type of PA	(Neuro) Peptide	Main Results
Controlled trial [38]	12 M Healthy Exercise status was not mentioned 19–29 years old No fasted state	Levels of plasma acylated ghrelin	Acute sprint interval and endurance exercise	Ghrelin	Sprint interval exercises lead to greater suppression in plasma acylated ghrelin levels.
Randomized crossover [39]	9 M Healthy Physically active 19–25 years old Fasted state	Levels of plasma acylated ghrelin	Acute aerobic exercises	Ghrelin	Single sessions of aerobic exercises lead to lower plasma levels of acylated ghrelin.
Randomized crossover [40]	11 M Healthy Physically active 19–23 years old Fasted state	Levels of plasma acylated ghrelin	Acute aerobic and resistance exercises	Ghrelin	Aerobic and resistance exercises lead to lower plasma levels of acylated ghrelin.
PA intervention, no control group [41]	5 M Healthy Sedentary 26 ± 1 years old Fasted state	Levels of plasma ghrelin	Acute incremental exercise until exhaustion	Ghrelin	Acute incremental exercise lads to decrease in plasma ghrelin levels.
Randomized crossover [42]	12 M overweight Sedentary 48 ± 5 years old Fasted state	Levels of plasma acylated ghrelin	Acute aerobic, strength and combined (aerobic and strength) exercises	Ghrelin	Single sessions of aerobic exercises lead to lower plasma levels of acylated ghrelin. Strength and combined exercises did not alter acylated ghrelin levels.
Randomized crossover [43]	18 M and F Healthy Physically active 19–32 years old Fasted state	Levels of total plasma ghrelin	Acute aerobic exercises	Ghrelin	Aerobic exercise did not alter total ghrelin levels.
Counter-balanced [44]	9 M Healthy Physically active 25 ± 1 years old Fasted state	Levels of plasma ghrelin	Acute resistance exercises	Ghrelin	Eccentric resistance exercises did not alter ghrelin levels and concentric resistance exercises suppressed ghrelin levels.
Randomized counter-balanced [45]	10 M Physically active 21 ± 1 years old Fasted state	Levels of plasma active ghrelin	Acute aerobic and resistance exercises	Ghrelin	Resistance exercises suppress plasma active ghrelin levels. Aerobic exercise did not alter plasma active ghrelin levels.
Longitudinal study [46]	22 M and F Overweight Sedentary 37 ± 8 years old Fasted state	Levels of plasma acylated ghrelin	Regular aerobic exercises	Ghrelin	Long-term aerobic training program results in slight increase in plasma acylated ghrelin levels.
RCT [47]	33 M overweight Sedentary 49 ± 7 years old Fasted and postprandial state	Levels of plasma active ghrelin	Regular aerobic and regular resistance exercises	Ghrelin	Aerobic and resistance exercises result in no change in acylated ghrelin levels in fasted and postprandial state.
Randomized trial [48]	20 M Healthy Sedentary 33 ± 2 years old Fasted state	Levels of plasma active ghrelin	Regular aerobic exercises in normoxic and normobaric hypoxic conditions	Ghrelin	4-week program of aerobic exercises in normoxic and normobaric hypoxic conditions result in no change in active ghrelin levels.

M, males; F, females; RCT, randomized controlled trial; PA, physical activity.

**Table 3:** Human studies on effects of physical activity on peptides and cognition: vasoactive intestinal peptide (VIP), neuropeptide Y (NPY), and calcitonin gene-related peptide GCRP.

Reference	Subjects	Target Area	Type of PA	(Neuro)Peptide	Main Results
Randomized PA intervention, no control group [49]	24 M Physically active 22–24 years old military cadets	Levels of plasma VIP	5-day period of strenuous exercise (35% of O2 uptake)	VIP	2 to 5-fold increase in plasma VIP, peak at day 2. Calorie-compensation and glucose infusion lowered the plasma VIP levels during the exercise period within 30-60 min of ingestion.
PA intervention, no control group [50]	6 M Exercise status was not mentioned 17–31 years old	Levels of plasma VIP	3 h of mild PA, short term-submaximal and maximal, fasting of 59 h	VIP	Increase of plasma VIP after 3 h of mild exercise and after fasting period. No elevation with short-term submaximal or maximal PA.
PA intervention, no control group [51]	18 M Age and exercise status was not mentioned Healthy	NPY release from the myocardial tissue during exercise	Supine position ergometer exercise	NPY	Small increase of NPY and NA release was detected during normoxic exercise, in hypoxic conditions 4 (NPY) and 2 (NA)–fold increases were detected.
PA intervention, no control group [52]	12 M Physically active 22 ± 3 years old elite rowers	Levels of plasma NPY	High-volume low intensity resistance training combined with endurance training for 2 weeks.	NPY	The post-exercise plasma NPY levels increased significantly with the 2-week training period but the levels normalized after 1-week of recovery.
PA intervention, no control group [53]	16 M Physically active 18–45 years old long distance swimmers	Levels of plasma NPY	25 km sea swimming competition (mean completion time 8,5 h)	NPY	Plasma NPY significantly increased. The level of plasma NPY app. doubled with swimming.
Between-group design [54]	10 F 66–81 years old And 6 M and F 21–46 years old Exercise status was not mentioned	Levels of plasma CGRP	Acute aerobic exercises	CGRP	Aerobic exercise increases the plasma CGRP levels.
PA intervention, no control group [55]	9 M Physically active 41–50 years old runners	Levels of plasma CGRP	Long-term aerobic exercises	CGRP	Aerobic exercise increases the plasma CGRP levels.

M, males; F, females.
